# Mobile Phone Intervention Reduces Perinatal Mortality in Zanzibar: Secondary Outcomes of a Cluster Randomized Controlled Trial

**DOI:** 10.2196/mhealth.2941

**Published:** 2014-03-26

**Authors:** Stine Lund, Vibeke Rasch, Maryam Hemed, Ida Marie Boas, Azzah Said, Khadija Said, Mkoko Hassan Makundu, Birgitte Bruun Nielsen

**Affiliations:** ^1^Department of International Health, Immunology and MicrobiologyUniversity of CopenhagenCopenhagen NDenmark; ^2^Institute of International HealthDepartment of International Health, Immunology and MicrobiologyUniversity of CopenhagenCopenhagenDenmark; ^3^Department of Obstetrics and GynecologyDepartment of Obstetrics and GynecologyOdense University HospitalOdenseDenmark; ^4^Reproductive and Child Health UnitMinistry of HealthRevolutionary Government of ZanzibarZanzibarTanzania, United Republic Of; ^5^Department of Obstetrics and GynecologyDepartment of Obstetrics and GynecologyAarhus University HospitalAarhusDenmark

**Keywords:** perinatal mortality, text messaging (SMS), mobile phones, developing countries

## Abstract

**Background:**

Mobile phones are increasingly used in health systems in developing countries and innovative technical solutions have great potential to overcome barriers of access to reproductive and child health care. However, despite widespread support for the use of mobile health technologies, evidence for its role in health care is sparse.

**Objective:**

We aimed to evaluate the association between a mobile phone intervention and perinatal mortality in a resource-limited setting.

**Methods:**

This study was a pragmatic, cluster-randomized, controlled trial with primary health care facilities in Zanzibar as the unit of randomization. At their first antenatal care visit, 2550 pregnant women (1311 interventions and 1239 controls) who attended antenatal care at selected primary health care facilities were included in this study and followed until 42 days after delivery. Twenty-four primary health care facilities in six districts were randomized to either mobile phone intervention or standard care. The intervention consisted of a mobile phone text message and voucher component. Secondary outcome measures included stillbirth, perinatal mortality, and death of a child within 42 days after birth as a proxy of neonatal mortality.

**Results:**

Within the first 42 days of life, 2482 children were born alive, 54 were stillborn, and 36 died. The overall perinatal mortality rate in the study was 27 per 1000 total births. The rate was lower in the intervention clusters, 19 per 1000 births, than in the control clusters, 36 per 1000 births. The intervention was associated with a significant reduction in perinatal mortality with an odds ratio (OR) of 0.50 (95% CI 0.27-0.93). Other secondary outcomes showed an insignificant reduction in stillbirth (OR 0.65, 95% CI 0.34-1.24) and an insignificant reduction in death within the first 42 days of life (OR 0.79, 95% CI 0.36-1.74).

**Conclusions:**

Mobile phone applications may contribute to improved health of the newborn and should be considered by policy makers in resource-limited settings.

**Trial Registration:**

ClinicalTrials.gov NCT01821222; http://www.clinicaltrials.gov/ct2/show/NCT01821222 (Archived by WebCite at http://www.webcitation.org/6NqxnxYn0).

## Introduction

With an increase in the number of mobile phone subscribers from 17 million in 2000 to 650 million in 2011, sub-Saharan Africa is experiencing a technological revolution [1]. The benefits of using mobile phone technology in health care systems in developing countries are diverse, and include improved reporting in health information systems, telemedicine providing care to populations otherwise deprived, and texting to improve adherence to treatment therapy [2,3]. Mobile health has great potential for sexual and reproductive health care and is seen as a key area in achieving the goals of the United Nations and the World Health Organization’s (WHO) Global Strategy for Women’s and Children’s Health [4]. Despite widespread support for the use of mobile health technologies, evidence for its role in health care is sparse [5]. We are unaware of any other cluster-randomized controlled trial that has assessed the use of a mobile phone intervention to improve perinatal survival in a resource-limited setting.

Perinatal death is among the most devastating adverse outcome of pregnancy. Over 2.65 million stillbirths and 3 million early neonatal deaths occur each year worldwide [6,7]. Of these deaths, 99% take place in low- and middle-income countries [8,9]. Although under-five mortality has declined over the past 25 years, there has been little progress in reduction of neonatal mortality and it is an increasingly prominent component of the overall rate of under-five mortality [10]. Therefore, to achieve Millennium Development Goal 4 (reducing child mortality), reducing perinatal mortality remains a challenge.

Perinatal mortality is closely linked to maternal mortality and causes of death are similar, often obstetric in origin, including prolonged labor, preeclampsia, infection, and obstetric hemorrhage [8,9]. The three major causes of neonatal death are infections, intrapartum related causes, and preterm birth complications, contributing approximately one-third each [10,11]. Stillbirth accounts for approximately one-half of all perinatal deaths. Antepartum stillbirths are associated with maternal infection, hypertension, and fetal growth restriction, while intrapartum stillbirth is associated with obstetric emergencies and lack of skilled care [8,12]. Perinatal mortality is a sensitive indicator of the quality of antenatal, obstetric, and early neonatal care available to women and newborns in any setting. Unfortunately, those women at greatest risk are least likely to have access to life-saving interventions and in low-income countries where resources are limited, antenatal care coverage is poor, and many women deliver at home without skilled attendance and newborn care is inadequate [13,14]. Zanzibar is similar to many other developing countries where little is known about perinatal mortality. According to the 2010 Demographic and Health Survey the perinatal mortality rate in Zanzibar is 50 per 1000, and 36 per 1000 in mainland Tanzania [15].

This report presents the detailed effect of a mobile phone intervention named *Wired Mothers* on the secondary outcomes stillbirth, perinatal death, and death of a child within the first 42 days of life. *Wired Mothers* links women to the health system throughout their pregnancy, childbirth, and postpartum period using a text message and free call voucher system. A cluster randomization with the primary health care facility as the unit of randomization was carried out to evaluate the intervention. The design was chosen to prevent contamination between women cared for at the same facility.

## Methods

### Design

The *Wired Mothers* study is a pragmatic, randomized, controlled trial with the primary health care facility as the unit of randomization. The study took place from 2009 to 2010 on the island of Unguja in Zanzibar, a semi-autonomous part of the United Republic of Tanzania. We followed the Consolidated Standards of Reporting Trials guidelines for reporting cluster-randomized trials [16].

### Participants

**Figure 1:**
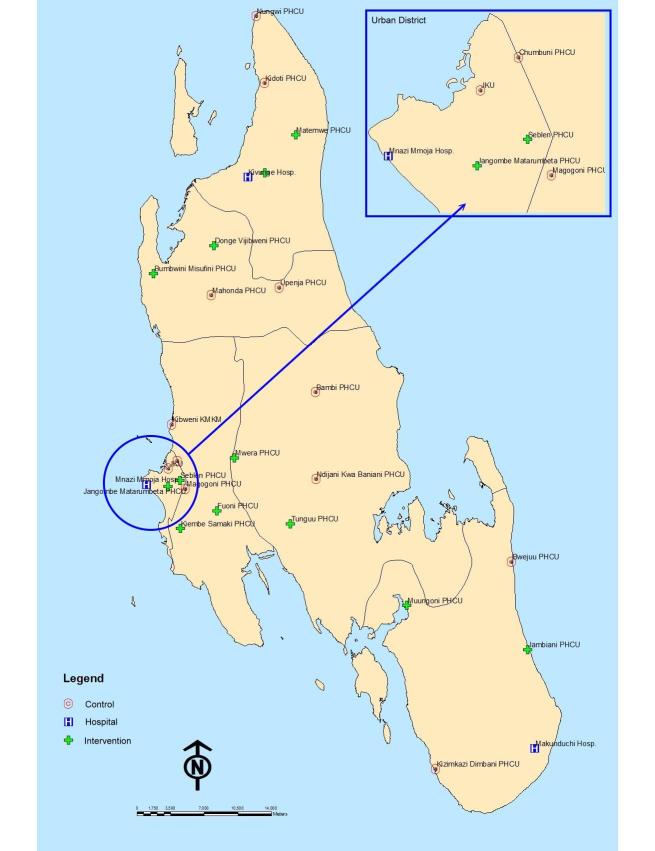
Research districts and location of intervention and control health facilities.

**Figure 2:**
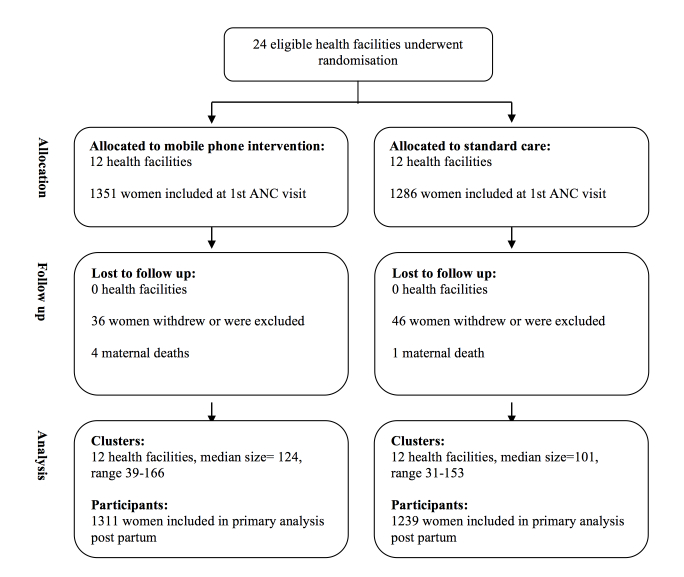
Procedures for the selection of the study population.

The study comprised 24 primary health care facilities and pregnant women attending antenatal care at these facilities. Clusters eligible for randomization were the four primary health care facilities in each of the six districts of Unguja Island with the most antenatal care visits in the previous year and a midwife among the staff. There were no major differences between included facilities. They were all primary health care facilities staffed with 1 or 2 midwives and access to basic infrastructure and equipment. The distribution of facilities in relation to hospitals providing Emergency Obstetric and Neonatal care was the same in intervention and control clusters (Figure 1). The eligibility criteria for participants was pregnant women who attended their first antenatal care visit at 1 of the 24 primary health care facilities regardless of gestational age or mobile phone ownership. A total of 2550 women were included in the study (Figure 2). Twenty-two women miscarried and 82 women withdrew or were not contactable during follow-up. Of these, 15 were known to have travelled outside the study area and three were not pregnant. During the study period 5 women died as a result of direct obstetric complications.

**Figure 3:**
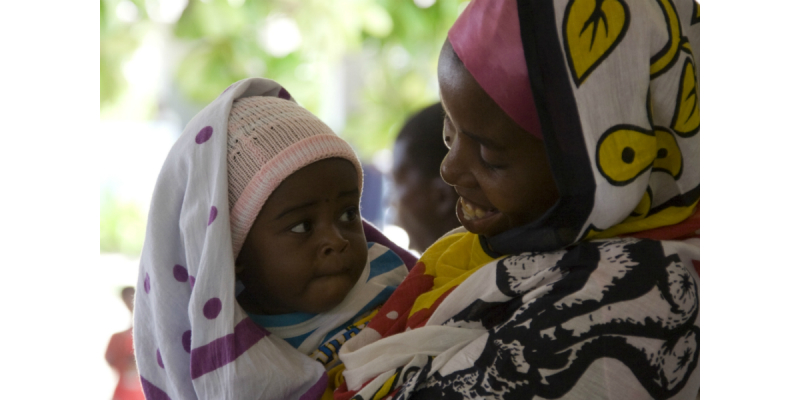
A Wired Mother with her child.

### Ethics

The Research Council of Zanzibar approved the study protocol on January 27^th^, 2009. The trial is registered with ClinicalTrials.gov, NCT01821222. All women were informed about the nature and purposes of the study as summarized in the consent form written in the local language, Swahili. All women provided their consent either by signature or fingerprint prior to their inclusion in the study. Women were free to drop out of the study at any time without a change in the quality of care provided. All study results and completed questionnaires were kept confidential and were not accessible to people outside of the research team. The trial is registered after enrollment of participants, due to researchers not being aware of this International Committee of Medical Journal Editors publication demand for relatively benign interventions without individual randomization such as the *Wired Mothers*.

### Intervention

The *Wired Mothers* mobile phone intervention was designed with the aim to link pregnant women to their primary health care provider throughout their pregnancy, childbirth, and postpartum period. The intervention was developed in Tanzania using simple technology and at low cost. It consists of two components: an automated short message service (SMS) system providing wired mothers with unidirectional text messaging and a mobile phone voucher system providing the possibility of direct two-way communication between wired mothers and their primary health care providers.

Women in the intervention group were registered at their first antenatal care visit with date, a phone number, and gestational age. The phone number was either their own or an access phone number of a husband/relative/friend. A specially-designed software that creates an individual pregnancy timeline for each woman and automatically sends text messages to the registered phone number was developed. The content and the frequency of the messages varied throughout the pregnancy and were intensified to weekly messages during the 4 weeks before delivery. The content of the messages focused on health education on topics, such as danger signs in pregnancy and the importance of skilled delivery attendance as well as appointment reminders for the next antenatal care visit. A total of 29,000 SMS were sent during the intervention period. Because the wired mothers intervention was developed in the context of the Ministry of Health in Zanzibar prioritizing to reduce maternal mortality, a voucher system was added to improve access to emergency obstetric care and improve referral mechanisms. Each intervention woman received the phone number of her local midwife and a small voucher of 500 TSH (Tanzanian shilling) allowing to call her. The women were not provided with mobile phones but a referral link was created in the health system through provision of mobile phones to midwives in primary health care facilities, and to midwives/doctors/drivers at the hospital level.

### Implementation

Twenty-four primary health care facilities were eligible for the study and the Ministry of Health agreed to let the facilities be included in the trial (cluster level consent). Meetings were held with staff in participating primary health care facilities to explain the nature and purpose of the trial. The enrolled primary health care facility staff also functioned as research assistants recording an inclusion questionnaire with demographic and covariate information, registering each contact with the women and completing an end-of-study questionnaire 6 weeks after delivery. Research assistants were assigned to the 3 hospitals providing emergency obstetric care and all contact with the enrolled women were similarly recorded. All pregnant women attending their first antenatal care visit in one of the participating primary health care facilities, if willing to participate, were included in the study. All enrolled women received an individual identity number and card. Pregnancy outcome was recorded at delivery for facility-based deliveries and for all included women in a follow-up interview 42 days after delivery. If the women did not return for the end-of-study interview, the research assistant contacted them either directly or by phone. Women attending the standard care received the protocols recommended in Zanzibar in the best format offered in these facilities. Double entry of data was performed in Epidata, transferred, and validated in SPSS.

### Outcomes

We evaluated the effect of a mobile phone intervention on the primary outcomes antenatal care (four or more visits) and skilled delivery attendance. These are presented in other papers [17,18]. Here, we present the interventions association with secondary outcomes stillbirth, perinatal death, and death of a child within the first 42 days of life. The intervention association with antenatal care and skilled delivery attendance is presented in other manuscripts. A perinatal death was defined as a composite of either a stillbirth or early neonatal death. We used the WHO agreed definition of stillbirth as any delivery in the third trimester (≥1000 g birth weight or ≥28 weeks of gestation) in which no signs of life (breathing, crying, heartbeat, movement) were evident [9]. An early neonatal death was defined as all babies born alive in the third trimester who die on or prior to day 7 after birth. The perinatal mortality rate is perinatal death per 1000 total births (live births and stillbirths). We included death of a child within 42 days (where end-of-study questionnaire was conducted) as a proxy of neonatal mortality.

### Sample Size

Power calculations were made on the primary outcomes skilled birth attendance and antenatal care attendance and did not take into account the clustering effect. Based on the number of antenatal care attendees from the previous year, the expected of size of the study population during a 3-month enrolment period was estimated to be 1100 women in the intervention group and 1375 women in the control group. Subsequently, a power calculation, based on data on antenatal care visits and skilled delivery attendance from the Tanzanian Demographic Health Survey (DHS 2005), was performed to document if the expected study population would be sufficient to document a true difference between the intervention and the control group [19]. To estimate whether this sample size was sufficient for detection of public health relevant effects of the intervention, we used data from the DHS 2005. For instance, with a 95% probability and a power of 90%, 894 women (447 in each group) were necessary for showing an increase of a relevant size (10% increase in the number of women delivering with a skilled birth attendant).  Hence, according to our power calculations, our proposed sample size was sufficient to document an effect of our intervention on antenatal care and skilled delivery attendance.

### Randomization and Blinding

Primary health care facilities, stratified by district, were assigned by simple random allocation to either the mobile phone intervention or control group (Figure 1). Clusters and study participants were not masked due to the nature of the intervention requiring overt participation.

### Statistical Analysis

Analyses were performed based on the “intention to treat” principle and all available data were included in the analysis. We adjusted for the clustering of our data using generalized estimating equations in all logistic regression analyses. We specified an exchangeable working correlation to allow for within cluster correlation and standard errors were based on the robust covariance matrix. We used the traditional logit link, which resulted in odds ratios (ORs) as an effect measure. However, for small values of the risk these can approximately be interpreted as relative risks. For our binary outcome measure, perinatal death yes/no, logistic multilevel analysis was used to analyze if there was a difference in perinatal deaths between the intervention and control groups. In this model, we included all socioeconomic and obstetric confounding variables and eliminated them using backward elimination (age, occupation, education, mobile phone status, residence, parity, previous caesarean section, multiple-gestation pregnancy). Variables with statistical significance were included in the final model. These were age and multiple-gestation pregnancy. Premature delivery, mode of delivery, four or more antenatal care visits, and delivery attendant were considered intermediate variables and not included in the model. We found no interaction between the intervention and explanatory variables. For other secondary outcomes we used a similar approach. Results were expressed as OR for perinatal deaths with 95% CI. Because perinatal mortality is a rare event this can be interpreted as a relative risk. For all models the criterion for significance was set at *P*<.05 and all analyses were performed using SPSS (version 20).

## Results

Socioeconomic characteristics of the study population were similar in intervention and control clusters. On average, mobile phones were owned by 37% of women, and 58% resided in rural areas (Table 1). Obstetric risk factors were also similar in the intervention and control clusters. On average, 7% of the women had previously had a caesarean section, 20% were pregnant for the first time, and 25% were in their fifth or more pregnancy (Table 2). Twin pregnancies accounted for 1%. Of these 15 pairs of twins, 2 were stillborn, and 4 were early neonatal deaths. In the studied pregnancies, 13.9% (182/1311) of intervention women and 16.1% (199/1239) of control women had a complication (Table 2). There was a difference in mode of delivery with more control women (29/1202, 2.4%) delivering with fundal pressure versus intervention women (3/1284, 0.2%). The cesarean section frequency was approximately the same with 3.5% (45/1284) of intervention and 3.8% (46/1202) of control women (Table 2). More women in the intervention group (574/1311, 43.8%) received the recommended four or more antenatal care visits than in the control group (385/1239, 31.1%). More intervention women delivered with skilled delivery attendance (766/1284, 59.7% vs 560/1201, 46.6%), and as previously described this difference was only statistically significant for urban women [17].

Overall, 2482 children were live born, 54 were stillborn, and 36 died within the first 42 days of life (Table 3). Of these, 69 children were perinatal deaths. Nine children were reported dead, but had an unknown time of death and they were, therefore, not included as perinatal deaths. The stillbirth rate was 17 per 1000 births in the intervention group versus 26 per 1000 births in the control group (Table 3). The overall perinatal mortality rate was 27 per 1000 total births. The rate was lower in the intervention clusters, 19 per 1000 births, than in the control clusters, 36 per 1000 births (Table 3). The intervention was associated with a significant reduction in the outcome perinatal mortality with an OR 0.50 (95% CI 0.27-0.93) (Table 4). Other secondary outcomes showed an insignificant reduction in stillbirths (OR 0.65, 95% CI 0.34-1.24) and an insignificant reduction in death of a child within 42 days (OR 0.79, 95% CI 0.36-1.74) (Table 4).

**Table 1:** Socioeconomic characteristics of study population.

Variable		Intervention	Control
		n (%)	n (%)
**Health facilities**			
	Number	12	12
**Participants**			
	Number of women	1311 (51.4)	1239 (48.6)
**Age** ^a^			
	<19	107 (8.5)	118 (9.9)
	20-24	310 (24.6)	307 (25.6)
	25-29	371 (29.5)	309 (25.8)
	30-34	248 (19.7)	259 (21.6)
	35+	222 (17.6)	204 (17.0)
**Occupation** ^b^			
	Housewife	691 (53.0)	691 (55.8)
	Farmer	286 (21.9)	241 (19.5)
	Sales women	133 (10.2)	117 (9.4)
	Government	51 (3.9)	46 (3.7)
	Student	22 (1.7)	19 (1.5)
	Other	121 (9.3)	112 (9.0)
**Education** ^c^			
	No	204 (16.0)	220 (18.3)
	Primary	464 (36.3)	440 (36.7)
	Secondary and above	569 (44.5)	503 (41.9)
	Other (religious education)	41 (3.2)	37 (3.1)
**Mobile phone status** ^d^			
	Owns	494 (37.8)	439 (35.5)
	Does not own	813 (62.2)	796 (64.5)
**Residence status**			
	Rural	743 (56.7)	730 (58.9)
	Urban	568 (43.3)	509 (41.1)

^a^Missing cases 95

^b^Missing cases 20

^c^Missing cases 72

^d^Missing cases 8

**Table 2:** Obstetric characteristics of study population.

Variable		Intervention	Control
		n (%)	n (%)
**Parity** ^a^			
	Prime	264 (20.5)	233 (19.4)
	1-2	428 (33.2)	356 (29.6)
	3-4	292 (22.6)	297 (24.7)
	5+	306 (23.7)	315 (26.2)
**Previous caesarean section** ^b^			
	Yes	72 (7)	69 (7)
	No	926 (93)	872 (93)
**Multiple-gestation pregnancy** ^c^			
	Multiple gestation	9 (0.7)	6 (0.5)
	Single gestation	1297 (99.3)	1231 (99.5)
**Premature delivery** ^d^			
	<37 gestation weeks	600 (46.3)	550 (45.9)
	At term	697 (53.7)	649 (54.1)
**Severe complication**			
	Yes	182 (13.9)	199 (16.1)
	No	1129 (86.8)	1040 (83.9)
**Mode of delivery** ^e^			
	Spontaneous vaginal	1231 (95.9)	1122 (93.3)
	Fundus pressure	3 (0.2)	29 (2.4)
	Assisted vaginal delivery	5 (0.4)	5 (0.4)
	Cesarean section	45 (3.5)	46 (3.8)
**Antenatal care**			
	Four or more visit	574 (43.8)	385 (31.1)
	Less than three visits	737 (56.2)	854 (68.9)
**Delivery attendant** ^**f,g**^			
	Skilled	766 (59.7)	560 (46.6)
	Unskilled	518 (40.3)	641 (53.4)

^a^Missing cases 59

^b^Missing cases 611

^c^Missing cases 7

^d^Missing cases 54

^e^Missing cases 62

^f^Missing cases 65

^g^We used the WHO definition, whereby skilled delivery attendants are midwifes, doctors, or nurses who have been educated and trained in the skills needed to manage pregnancies, childbirth, and the immediate postnatal period, including the identification, management, and referral of complications in women and newborns. We also included home deliveries assisted by skilled birth attendants, although international consensus has not been reached on this issue.

**Table 3:** Number of births, deaths, and mortality rates.

Variable		Intervention	Control	Total
		n	n	n
Total births		1300	1236	2536
Live birth		1278	1204	2482
**Still birth**		22	32	54
	Fresh	17	24	41
	Macerated	5	8	13
Perinatal mortality		25	44	69
Neonatal mortality^a^		18	18	36
Still birth rate (per 1000 births)		17	26	21
Perinatal mortality rate (per 1000 births)		19	36	27
Neonatal mortality rate^b^ (per 1000 live births)		14	15	15

^a^Missing cases 7

^b^Death<42 days

**Table 4:** *.* Intervention association with secondary outcomes.

Variable		Unadjusted OR^a,b^ (95% CI)	Adjusted OR^a,c^ (95% CI)
**Stillbirth**			
	Intervention vs control	0.62 (0.31-1.22)	0.65 (0.34-1.24)
**Perinatal mortality**			
	Intervention vs control	0.49 (0.27-0.90)	0.50 (0.27-0.93)
**Neonatal mortality** ^d^			
	Intervention vs control	0.85 (0.37-1.95)	0.79 (0.36-1.74)

^a^Odds ratio

^b^Adjusted for within-cluster effect

^c^Adjusted for within-cluster effect and significant variables associated with perinatal mortality

^d^Death<42 days

## Discussion

### Principal Findings

Our findings showed an association between the *Wired Mothers* mobile phone intervention and a reduction in perinatal mortality. Children born by women in the intervention group had a 50% reduction in perinatal mortality compared with children born by women in the control group. There was an insignificant reduction in death of children within the first 42 days, indicating that the beneficiary implications of the *Wired Mothers* intervention was centered on improving women’s choices of care and access to care during pregnancy and in the time surrounding the delivery.

### Strengths and Limitations

The principal strength of our study is that it met the requirement of systematic reviews calling for trials of mobile phone interventions in developing countries [2,3]. The intervention was developed at low cost in Tanzania using locally-based software development expertise to promote sustainability of the program. We used a robust study design with a relatively large sample of health facilities and participants. Others have used similar design for trailing complex interventions in low-resource settings [20]. Intervention and control clusters were similar in socioeconomic and obstetric background characteristics, and it is reassuring that after adjustment for identifiable potential risk factors, the crude and adjusted results are similar. Blinding was impossible due to the nature of the intervention increasing the chance of a selection or information bias. Because 9 children had an uncertain time of death there is a risk that we may have underreported perinatal deaths in either intervention or control clusters. It is a limitation that the number of women with miscarriages (22) or who withdrew from the study (82) exceeds those with the outcome perinatal death (69), particularly because there is a high likelihood of worse outcomes in those women lost to follow up. We therefore caution that other studies with perinatal mortality as a primary outcome should confirm our findings.

### Comparison With Other Studies

Because this is the first trial assessing the association between a mobile phone intervention and perinatal mortality we cannot compare with other results. Free et al [2,3] found that while mHealth studies have been conducted many are of poor quality, few have a low risk of bias, and very few have found clinically significant benefits of the interventions. The 2012 Lancet report of technologies for global health identified only nine randomized controlled trials for mHealth in low-income countries [21].

Our results are in line with other studies of perinatal mortality in sub-Saharan Africa, although the perinatal mortality rate for our study (27 per 1000 births) was below the estimates for Tanzania [6,9,22]. Our study confirms results from others indicating that stillbirths may constitute up to 70%-80% of perinatal deaths and underlines the importance of access to good quality antenatal and delivery care [22]. Being one of the main causes of early neonatal death it is a concern that we found higher incidences of prematurity than otherwise reported from the region [23,24]. The gestational age at delivery is however based on provider estimates and should be interpreted with caution. It is apparent that with access to adequate medical care, especially in the intrapartum and early neonatal periods, many perinatal deaths might be prevented [25]. For instance, a study from Tanzania assessed a basic neonatal resuscitation training at hospital level and demonstrated a remarkable sustained 47% reduction in early neonatal mortality (within 24 hours) and a 24% reduction in fresh stillbirths [26]. The authors suggest that the most plausible explanation for this reduction in fresh stillbirths was that most nonbreathing infants are in primary apnea with a heart rate and will initiate spontaneous respiration in response to drying and stimulation only if implemented in a timely manner [26]. We found an association between the *Wired Mothers* intervention and a reduction in perinatal mortality. However, we cannot explain the exact factors that contributed to this. There was an increase of antenatal care and skilled delivery attendance in the intervention clusters but, for instance, a low rate of caesarean section in both groups [17,18]. Hence, an increased level of caesarean section in the intervention group did not contribute to the reduction.

A major part of the evidence for sexual and reproductive mHealth comes from the use of text reminders. Studies primarily indicate potential to improve knowledge and awareness [5,27,28]. Another area where mHealth is widely used is text-based appointment reminders. Two recent reviews have found moderate quality evidence that mobile phone text message reminders for health care appointments are more effective than no reminders and that such interventions may be appropriate for implementation [2,29]. Similarly, our study has produced evidence of increased skilled delivery attendance among urban women benefiting from a mobile phone intervention (OR 5.73, 95% CI 1.51-21.81) and an overall increase in women attending antenatal care four or more times as recommended by the WHO (OR 2.39, 95% CI 1.03-5.55) [17,18]. There is, to our knowledge, only one other trial from a developing country that has attempted to use a health outcome as a primary outcome. In Kenya, a high quality trial used text messages to improve adherence to antiretroviral therapy among HIV-positive patients. This intervention significantly reduced the patients’ viral load but did not significantly reduce mortality [30]. Our results and the study from Kenya are in accordance with the limited existing literature on mHealth as a moderate tool for behavioral change, and indicate that positive behavioral effects can be reached in developing countries [2,3,31]. It is not possible to determine which component (eg, the SMS reminders or the vouchers) had the most significant impact on perinatal mortality. The *Wired Mothers* intervention sought to influence and improve all stages in receiving adequate care as proposed in the "Three Delays" model, and most notably the decision making to seek care and reaching an appropriate obstetric facility [32]. The SMS text messages aimed to empower women to make informed decision about attending regular antenatal care and delivery with a skilled attendant. The level of voucher contacts between the women and their primary health care providers were higher than expected. It served to improve emergency referrals, but it also served to increase linkages and trust on a more general basis.

### Policy Implications

The policy implications for this study are that text-based mobile phone interventions such as *Wired Mothers* should be considered to reduce perinatal mortality and to achieve Millennium Development Goal 4. An increasing number of developing countries have a framework in place with national mHealth policies and there are a number of mHealth solutions being piloted in developing countries. Few are being scaled up and the real test for mHealth is scalability and integration into existing systems [33]. Despite the global progress in reducing deaths of children younger than 5 years, there is little reduction in the global perinatal mortality rates. The causality of maternal deaths, stillbirths, and early neonatal deaths are interlinked. Therefore, monitoring and policies should be integrated. We suggest to include reduction of perinatal mortality in the post 2015 agenda to further reduce child mortality in developing countries.

### Conclusions

In conclusion, the *Wired Mothers* mobile phone intervention was associated with a reduction in perinatal mortality. Mobile phone applications may contribute to improved health of the newborn and should be considered by policy makers in resource-limited settings. Overall, there is limited evidence on the effects of mobile phone interventions and further high-quality research is required to draw more robust conclusions, particularly for developing countries within the field of reproductive and child health.

## Multimedia Appendix 1

The story of Jina - a wired mother.

## Abbreviations

DHS: Demographic Health Survey
OR: odds ratio
SMS: short message service
TSH: Tanzanian shilling
WHO: World Health Organization
